# Antibody kinetics and shedding dynamics of MERS-CoV in dromedary camels from different production systems in Kenya: a longitudinal cohort study

**DOI:** 10.1007/s11250-026-04981-3

**Published:** 2026-03-12

**Authors:** Rinah Sitawa Wangila, Thomas Nyariki, Dickson Machira Nyaguthii, Timothy Muthui Wachira, Joshua Kimutai, Allan Azegele, Romona Ndanyi, James Ngoci, Bridgit Muasa, George Njogu, Peter Baaro Gathura, Emma G. Gardener, El Masry Ihab, Joseph Kamau, Robert Ofwete, Chitwambi Makungu, Aminu Shittu, Charles Bebay, Folorunso O. Fasina

**Affiliations:** 1 Food and Agriculture Organization of the United Nations (FAO), Nairobi, Kenya; 2 Department of Public Health Pharmacology & Toxicology, University of Nairobi, Nairobi, Kenya; 3 Health Program, International Livestock Research Institute, Nairobi, Kenya; 4 Ministry of Agriculture and Livestock Development, Nairobi, Kenya; 5 Food and Agriculture Organization of the United Nations (FAO), Rome, Italy; 6 One Health Centre, Kenya Institute of Primate Research, Karen, Nairobi, Kenya; 7 Amref Health Africa, Nairobi, Kenya; 8 Food and Agriculture Organization of the United Nations (FAO), Lusaka, Zambia; 9 Department of Theriogenology and Animal Production, Faculty of Veterinary Medicine, Usmanu Danfodiyo University, Sokoto, PMB, Sokoto State 2254 Nigeria; 10 Department of Veterinary Tropical Diseases, Faculty of Veterinary Science, University of Pretoria, Onderstepoort, 0110 South Africa; 11 Present Address: Hagren One Health Consultants Ltd., Nairobi, Kenya

**Keywords:** Middle East respiratory syndrome coronavirus, Retrospective review, Antibody titres and nasal shedding dynamics

## Abstract

Understanding long-term antibody kinetics in different camel production systems is crucial for risk assessment for Middle East respiratory syndrome coronavirus (MERS-CoV), a zoonotic pathogen first detected in humans in Saudi Arabia. Though the virus is endemic in dromedary camels across Africa, the Middle East, and parts of South Asia, zoonotic transmission outside of the Arabian Peninsula is undocumented. A retrospective analysis of longitudinal surveillance data of MERS-CoV was conducted on 2460 serum samples collected from 174 camels in three high-risk counties of Kenya from April 2018 to March 2021. Data were analyzed using descriptive statistics, logistic regression, and survival analysis. A total of 93/174 (53.4%) camels were seropositive at least once (animal-level), while 882/2460 (35.85%) of all serum samples tested seropositive (sample-level). Data were analyzed using descriptive statistics, logistic regression and survival analysis. A total of 93/174 (53.4%) camels were seropositive at least once. All camels in the pastoral system seroconverted, exhibiting significantly higher odds of seropositivity (OR: 9.4) compared to ranched camels, and camels above 3 years (OR: 21.2) are more likely to be seropositive compared to young ones. The median duration of seropositivity was 21 days (IQR: 11–53), varying significantly by geographical site. Camels from Garissa and Soysambu have 97% (aOR: 0.03 (CI_95%_: 0.001–0.79; *p* = 0.035) and 98% (aOR: 0.02 (CI_95%_: 0.001–0.48; *p* = 0.015) longer durations of seropositivity than those from Isiolo. The study demonstrates the spatiotemporal and biological variability of antibody levels, indicative of fluctuations in viral exposure and the resultant immune response among the camel herds. Thus, camel production practices, not just seroprevalence, drive MERS-CoV infection dynamics, demanding surveillance and stewardship strategies that are tailored to specific production systems to effectively mitigate zoonotic risk.

**Supplementary Information:**

The online version contains supplementary material available at https://doi.org/10.1007/s11250-026-04981-3.

## Introduction

The Middle East respiratory syndrome coronavirus (MERS-CoV) was first isolated from a patient in Saudi Arabia presenting with severe pneumonia in June 2012 (Mohd et al. 2016). This lethal zoonotic pathogen, now endemic in the Arabian Peninsula, has since been detected in cases and camels across Europe, Africa, Asia, and North America (Azhar et al. 2014) (Zaki et al. 2012). As of 6th October 2025, the World Health Organization has reported 2,640 laboratory-confirmed human cases from 27 countries, including 958 deaths (“European Centre for Disease Prevention and Control,” 2025). Dromedary camels are the established zoonotic reservoir, with genetic studies confirming near-identical viruses in both camels and humans in shared localities (Younan et al., 2016). In Kenya, cross-sectional studies have confirmed the virus’s circulation in camels, with a national seroprevalence of 62.9%, varying from 77.7% in Isiolo County to 14.0% in Nakuru County (Sitawa et al. 2020).

The high case fatality rate (~ 36%) and the vast geographical range of the camel reservoir have led the WHO to classify MERS-CoV as a high-priority pathogen under its *Research and Development Blueprint* (Kelly-Cirino et al. 2019) Critically, while no human cases have been officially documented in Kenya, phylogenetic evidence suggests a history of undetected spillover events (Stalin Raj et al. 2018). While existing seroprevalence studies provide a snapshot of exposure, they fall short of characterizing the ongoing infection dynamics necessary for risk assessment. Although cross-sectional studies have established the presence of MERS-CoV in Kenyan camels, longitudinal data on antibody kinetics remain scarce. It remains poorly understood how antibody persistence and reinfection risks vary across the different management practices that define camel husbandry in Kenya, such as nomadic pastoralism versus commercial ranching. This study aimed to fill this critical gap by conducting a longitudinal analysis to (i) characterize MERS-CoV antibody kinetics and nasal shedding dynamics, and (ii) determine the association between seropositivity and key epidemiological factors including geographical location, production system, age, and sex. This was achieved through a systematic analysis of data generated from three longitudinal surveillance studies conducted in Kenya from 2018 to 2021.

## Materials and methods

### Study area and design

This study was designed as a series of longitudinal surveillance efforts conducted in three distinct phases across camel production systems in Kenya from April 2018 to March 2021. Each phase was implemented in a different geographical setting (Soysambu ranch in Nakuru County, and pastoral herds in Isiolo and Garissa counties) to capture the ecological and management variability inherent to MERS-CoV transmission dynamics in dromedary camels. While the phases differed in location, herd composition, and follow-up duration, all were harmonized through standardized protocols for sample collection, laboratory testing, and data recording. Serum and nasal swab samples were collected at approximately 10-day intervals across all phases, and serological and molecular assays were performed using consistent methodologies and cut-off criteria.

To ensure analytical rigor and address potential heterogeneity introduced by the multi-phase design, we adopted a stratified analytical approach. Analyses were conducted both within and across phases, with key variables—including production system, geographical site, and study phase—incorporated as stratification factors or covariates in regression and survival models. Where appropriate, clustering at the herd and phase levels was accounted for in statistical models to mitigate bias and preserve internal validity. This harmonized yet stratified approach allowed us to integrate data across phases while respecting contextual differences, thereby enhancing the external validity of findings and providing a more robust representation of MERS-CoV dynamics across diverse camel husbandry systems in Kenya.

The camel sera were collected during longitudinal follow-up studies conducted in three MERS-CoV high risk counties of Nakuru, Isiolo and Garissa from April 2018 to March 2021 (“*Risk Factors Predisposing Dromedary Camels and Pastoral Communities to Zoonotic.Pdf*,” 2023).

**Fig. 1:**
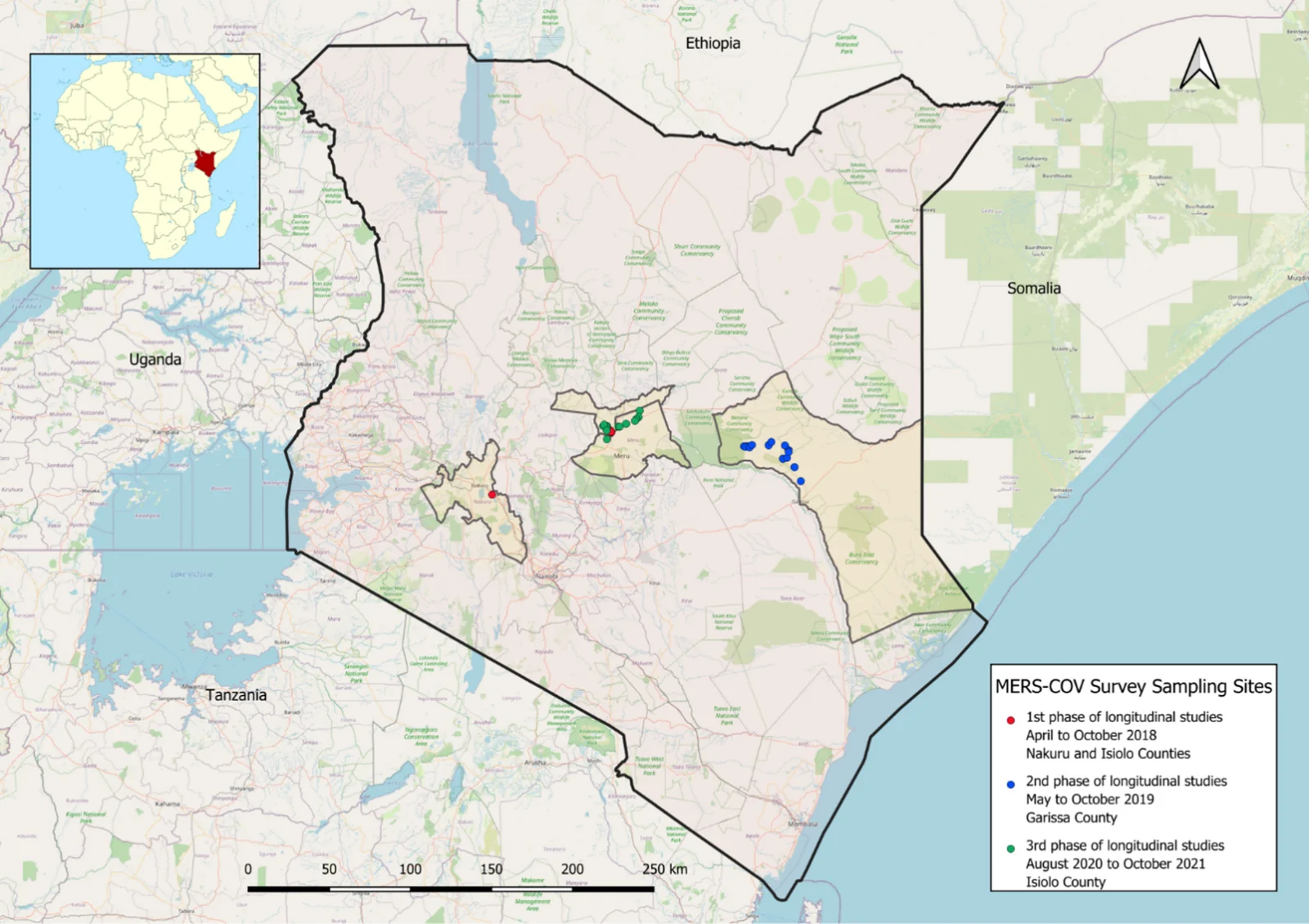
MERSCOV survey sampling sites

The 1st phase of longitudinal surveillance was conducted in Soysambu ranch, Nakuru County and a pastoral herd in Burat location of Isiolo County from April to October 2018. The 2nd phase of the study was conducted in a pastoral herd in Shimbrey location, Balambala Sub County, Garissa County from May to October 2019. The 3rd phase was conducted in a pastoral herd in Isiolo County, Burat location from August 2020 to October 2021 (Fig. 1).

### Sampling

In Isiolo county, 48 camels were recruited from a pastoral herd in Burat location while in Nakuru County, 50 camels from Soysambu ranch were recruited. These camels were sampled every 10 days. Additional samples were collected during the 2nd phase of the study conducted in a pastoral herd of 21 camels in Shimbrey location, Garissa County. The last batch of samples were collected in the 3rd phase of the study from a large camel pastoral herd in Burat location in Isiolo County from August 2020 to October 2021. This herd, different from the herd recruited in the 1st phase, constituted 27 calf dam pairs resulting in 54 camels sampled during the baseline. From the 2nd to the 11th round of sampling only the calves were sampled as compared to rounds 12 to 19 where the dams – calf pairs were sampled.

Serum and nasal swabs were collected according to the revised FAO swab and tissue sample collection procedures for enhancing MERS-CoV detection in camels and the laboratory guidelines (FAO, 2019). The nasal swabs and tissue samples in VTM and Trizol were placed in a liquid nitrogen (LN2) transport canister and transported to the National Veterinary Referral Laboratory (NVRL) in Kabete where the samples were stored till laboratory processing and testing.

### Laboratory analysis

Sera were tested for the presence of IgG antibodies reacting with MERS-CoV using the Anti-MERS-CoV ELISA Camel (IgG) protocol (EUROIMMUN, Medizinsche Labordiagnostika AG, 2015) and according to the manufacturer’s instructions. Camel sera were classified based on serostatus as determined by ELISA optical density readings that were converted to a ratio using the OD calibrator. ELISA test results were interpreted according to the kit’s manufacturer’s guidelines (cut-off ratios of ≤ 0.8 be interpreted as negative, ≥ 0.8 and ≤ 1.1 as borderline, and ≥ 1.1 as positive). Borderline results were classified as positive to maximize sensitivity for exposure detection, consistent with a precautionary approach in surveillance of emerging zoonoses. This may slightly inflate seroprevalence estimates but reduces the risk of missing true exposures. Samples collected in Garissa county in 2019 were additionally tested using a using a MERS-spike pseudo particle neutralization assay as described (Perera et al. 2013) and extensively validated (Hemida et al. 2013) (Park et al. 2015).

Hydrolysis probe–based real-time reverse transcription PCR (RT-PCR) targeting upstream of E gene of MERS-CoV was used for screening. Two targets, one targeting upE and the other ORF1a gene were used for confirmation as recommended by the World Health Organization (Hemida et al., 2017).

### Data management and analysis

Level of analysis clarification: Throughout this study, results are reported at the sample level (individual serum or swab results), animal level (per camel), or episode level (seropositivity episodes) as appropriate. Each table and results section explicitly states the level of analysis to ensure clarity. Data analysis was performed using Stata v15 (StataCorp., 2017). The outcome of interest for prevalence analysis was the ELISA result, categorized as negative or positive (with borderline results considered positive). Chi-square and Fisher’s exact tests were used to assess associations between categorical variables (e.g., production system, location, sex, age) and seropositivity.

To analyze the persistence of the antibody response, we calculated the duration of seropositivity episodes. An episode of seropositivity was defined as a period beginning with a positive test and ending with a negative test, allowing for a maximum gap of 14 days between consecutive positive results. The 14-day gap was chosen based on known shedding and seroconversion dynamics of MERS-CoV in camels and aligns with similar longitudinal studies (Munywoki et al. 2014). The median duration of seropositivity and Inter Quartile Range (IQR) were calculated from these episodes (Munywoki et al. 2014). To ensure accuracy, episodes that were ongoing at the start of the study (left-censored) or at the end of the sampling period (right-censored) were excluded from this specific calculation. Kaplan-Meier graphs were used to visualize seropositivity duration. A Cox proportional hazards model was used to investigate factors affecting the duration of seropositivity. Prior to model fitting, the proportional hazards assumption was assessed using Schoenfeld residuals. Where violations were detected, alternative survival models (stratified Cox or parametric models) were considered. (Nyaguthii et al. 2021). To determine risk factors for MERS-CoV seropositivity, the longitudinal serological data for this study were extracted from a comprehensive Excel dataset (“*MASTER TEMPLATE_27.7.22_C_Consolidated (2).xlsx*”) containing results from three sequential study phases conducted between 2018 and 2021. The original dataset employed a wide format structure, with each row representing an individual camel and columns capturing repeated ELISA and PCR measurements across seventeen collection time points. To prepare these data for longitudinal analysis, we restructured the dataset from wide to long format using Python (version 3.9) with the pandas library (version 1.4). This transformation involved extracting time-invariant variables (Camel ID, County, Age category, and Sex) alongside time-varying ELISA values for each collection point. ELISA measurements were converted to binary seropositivity outcomes using a validated cutoff of 0.5 optical density units. Based on geographical location, camels were classified into two production systems: “Ranched” (SOYSAMBU county) and “Pastoral” (ISIOLO and GARISSA counties). Data quality assurance procedures included excluding missing values (coded as “xxx” or blank), standardizing age categories across study phases, and verifying the uniqueness and consistency of camel identifiers.

The model was specified with seropositivity as the binary outcome at each sampling point (sample-level), and predictors included time (collection number), age group, sex, and production system. Camel ID was used as the clustering variable to account for repeated measures within animals. We employed Generalized Estimating Equations (GEE) with a logistic link function. The model was specified with seropositivity as the binary outcome, and predictors included time (collection number, treated as continuous), age group (categorical: 3–6 months, 6–12 months, 1–2 years, > 3 years), sex (binary: Male, Female), and production system (binary: Ranched, Pastoral). An exchangeable correlation structure was assumed, with camel ID as the clustering variable to account for within-animal correlation. Robust variance estimators were used to ensure valid inference even under potential misspecification of the correlation structure. Reference categories were set as follows: 3–6 months for age, Female for sex, and Pastoral for production system.

The analysis was implemented in Python using the statsmodels library (version 0.13) with a binomial family and logit link. Model convergence was achieved within 50 iterations using maximum quasi-likelihood estimation. Preliminary diagnostics revealed near-perfect prediction in the Pastoral system (96.8% seropositivity), which we addressed through robust variance estimation and sensitivity analyses. Missing data were minimal (< 5%) and handled via complete-case analysis under the assumption of missing completely at random. Model fit was assessed through examination of convergence stability and standard error reliability.

Results are presented in Table 3 as odds ratios with 95% confidence intervals and corresponding p-values, with significance levels indicated conventionally (*p < 0.05, **p < 0.01, ***p < 0.001). All analytical code is documented and available upon request to ensure transparency and reproducibility. Contextually, a camel that tested positive at least once during the study period was considered positive and if it tested negative throughout the whole study period it was considered negative, and this was used as the outcome variable tested against the predictor variables (production system, geographical site, gender, and age of the camels).

### Ethical approval and consent

Ethical approval for this study was inherent to the official sanitary mandate and approved national work plans (2018–2021) of the State Department of Livestock within the Ministry of Agriculture and Livestock Development, Nairobi, Kenya. As this surveillance was conducted as part of the Department’s statutory duty to monitor and control animal diseases for public health protection, a separate ethical review committee approval was not required under national regulations. All animal handling and sampling protocol followed FAO and WOAH guidelines for animal welfare, and that procedures were overseen by government veterinary officers under the national animal health regulatory framework, and aligned with international standards for veterinary surveillance studies.

Informed consent was obtained from all camel owners prior to sample collection. The purpose of the surveillance, the procedures involved, and the use of the data were explained verbally, and consent was documented by the attending government veterinary officers as part of the official fieldwork records.

## Results

### Herd demographics and characteristics

The demographic characteristics of the camel cohorts involved in the longitudinal study of Middle East respiratory syndrome coronavirus (MERS-CoV) in Kenya, from April 2018 to March 2021, are presented in Table 1.

**Table 1:** Demographic characteristics of camel cohorts (animal-level) in MERS-CoV longitudinal studies, 2018–2021, Kenya

The 1st phase					The 2nd Phase			The 3rd Phase		
**Variable**	Soysambu (Ranch)* (n = 51)		**Isiolo (Peri-urban Semi-Pastoral)** **(n = 49)**		**Variable**	**Garissa (Pastoral)** **(n = 21)**		**Variable**	**Isiolo (Peri urban Semi-pastoral)** **(n = 53)**	
	**Number**	**Percentage**	**Number**	**Percentage**		**Number**	**Percentage**		**Number**	**Percentage**
**Gender**										
Male	22	43.0	9	18.0	Male	1	5.0	Male	6	11.3
Female	29	57.0	40	82.0	Female	20	95.0	Female	47	88.7
**Age category (months)**										
12–24	12	24.0	30	61.0	1–6	2	9.5	1–6	4	7.5
> 24	15	29.0	13	27.0	7–24	2	9.5	7–24	23	43.4
< 12	24	47.0	6	12.0	> 24	17	81.0	> 24	26	49.1

Study 1 = April – October 2018; Study 2 = May – October 2019; and Study 3 = August 2020 – October 2021. The numbers indicated the number of camels sampled. Repeated-measure sampling was conducted up to 19 rounds in some herds. *Soysambu is in Nakuru County

### MERS-CoV Seropositivity and antibody kinetics

A longitudinal collection of 2460 serum samples from 174 camels revealed an overall sample-level MERS-CoV seropositivity rate of 35.85% (882/2460). At the animal level, 93 camels (53.4%) tested positive at least once. On average, each camel contributed 14.1 serum samples over 18 sampling sessions, with an average interval of 11.9 days between sessions (Table 2).

Seropositivity varied dramatically by production system and study site (Table 2). The pastoral production system in Garissa exhibited near-universal and persistent infection at the animal level, with 100% of camels (21/21) seroconverting, and a sample-level seropositivity rate of 89.5% (187/209) across samples. In contrast, the ranched system in Soysambu showed minimal viral circulation at the animal level, with only 21.6% of camels (11/51) ever testing positive, and a sample-level seropositivity of just 7.9% (62/786) (Table 2).

**Table 2:** Sample-level seropositivity of camel samples, 2018–2021, Kenya

Item	Category	Number of serum samples (%) *N* = 2460	Number of samples seropositive *n*/*N* (%).
Study site	Isiolo (2018)	828 (33.7)	448/828 (54.1)
	Soysambu (2018)	786 (32.0)	62/786 (7.9)
	Garissa (2019)	209 (8.5)	187/209 (89.5%)
	Isiolo (2021)	637 (25.9)	185/637 (29.0)
Gender	Male	570 (23.2)	28/570 (4.9)
	Female	1890 (76.8)	854/1890 (45.2)
Age	Newborn − 2 months	38 (1.5)	10/38 (26.3)
	3–6 months	204 (8.3)	3/204 (1.47)
	6–12 months	681 (27.7)	12/681 (1.8)
	1–2 years	313 (12.7)	31/313 (9.9)
	2–3 years	215(8.7)	94/215 (43.7)
	> 3 years	1009 (41.0)	732/1009 (72.6)
Production system	Pastoral	209 (8.5)	187/209 (89.5)
	Peri-urban semi-pastoral	1465(59.6)	633/1465 (43.2)
	Ranched	786 (32.0)	62/786(7.9)

Note: All proportions are sample-level (n/N samples). Animal-level seroprevalence is reported in the manuscript

Infection patterns were strongly associated with age, with seropositivity increasing significantly in older camels. While young camels (3–12 months) had very low seropositivity rates (1.5–1.8%), the rate rose to 9.9% in 1–2 year-olds and peaked at 72.6% in camels over 3 years old. Furthermore, female camels were significantly more likely to be seropositive (45.2% of samples; 62.0% of individuals) than males (4.9% of samples; 21.6% of individuals) (Table 2).

### Generalized estimating equations (GEE) model on the ELISA interpretation

The generalized estimating equations (GEE) analysis revealed that that production system and age were significant predictors of MERS-CoV seropositivity (Table 3). The odds of being seropositive were 8.3 times lower (95% CI: 0.08–0.18; *p*< 0.001) for ranched camels compared to camels under the pastoral system. Camels in the pastoral system have a perfect prediction of 97% seropositivity. Additionally, camels aged over 3 years were 2.18 times more likely (95% CI: 1.63–2.91; *p*< 0.001) to be seropositive compared to camels, 3–6 month old. Time per collection and sex of animal were not significant predictors in the GEE model (Table 3).

**Table 3:** Factors associated with MERS-CoV seropositivity using Generalized Estimating Equations (GEE) analysis

Factor	Category	Odd Ratio (95% CI)	*P*-value
Time per collection	Continuous variable	1.02 (0.98–1.06)	0.342
Age group	ref: 3–6 months	1.00	NA
	6–12 months	1.15 (0.82–1.61)	0.421
	1–2 years	1.42 (1.05–1.92)*	0.024
	> 3 years	2.18 (1.63–2.91)**	< 0.001
Sex	Ref: female	1.00	NA
	Male	0.89 (0.67–1.18)	0.414
Production system	ref: Pastoral	1.00	NA
	Ranched	0.12 (0.08–0.18)**	< 0.001

Perfect prediction in Pastoral system (97% seropositive), NA = Not applicable. Ref = reference.These results are reported at the sample level (individual serumresults)

### Median duration of seropositivity

The analysis of seropositivity episodes (episode-level) revealed that, at the animal level, 46.6% (81/174) of camels never seroconverted, while 42.5% (74/174) experienced a single episode. A minority experienced two (7.5%) or three (3.5%) episodes. Of the 118 total episodes identified, the majority (74.6%) were censored.

**Fig. 2:**
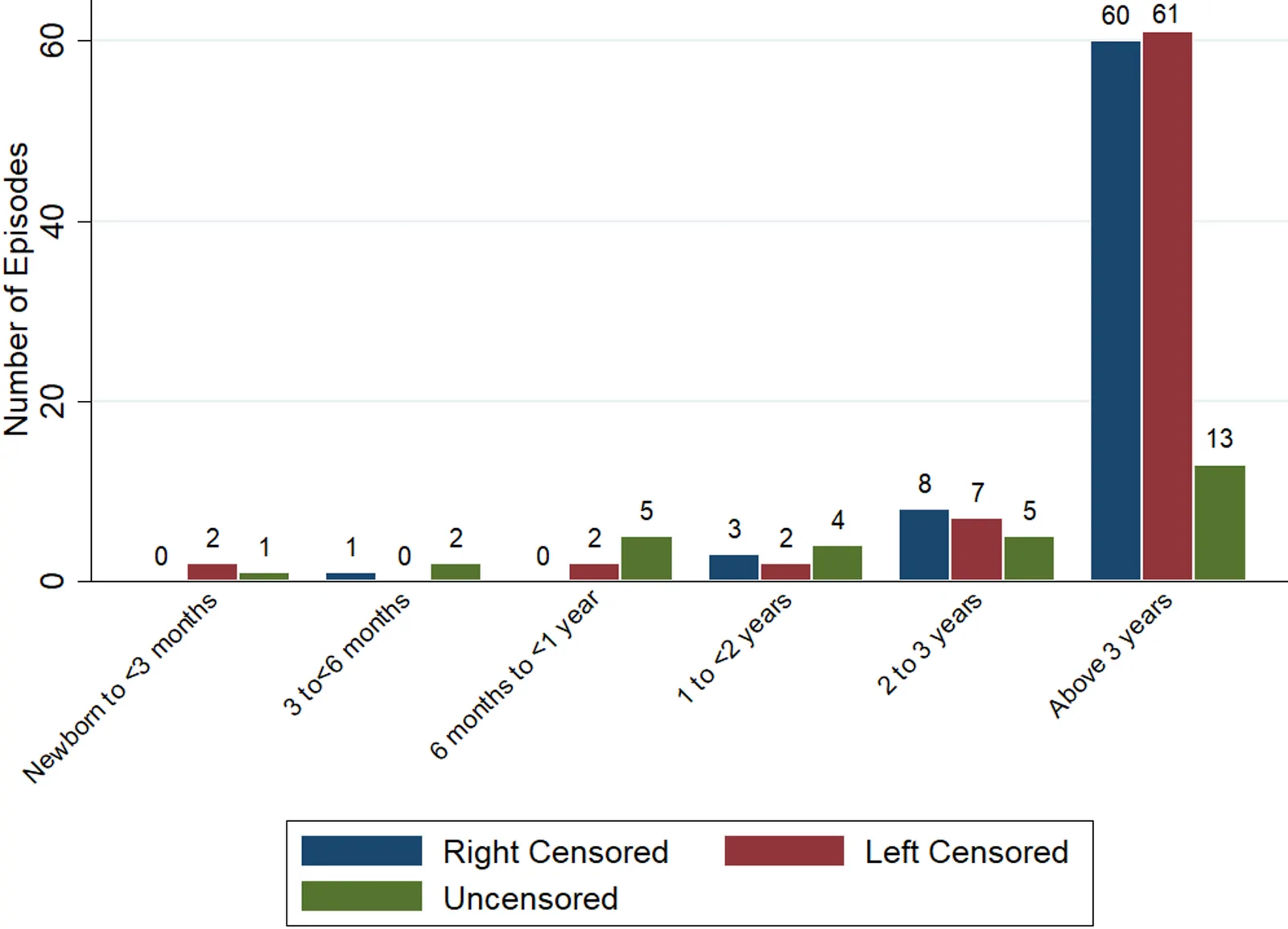
Episodes grouped by age. The episodes are characterized by their censoring status

**Fig. 3:**
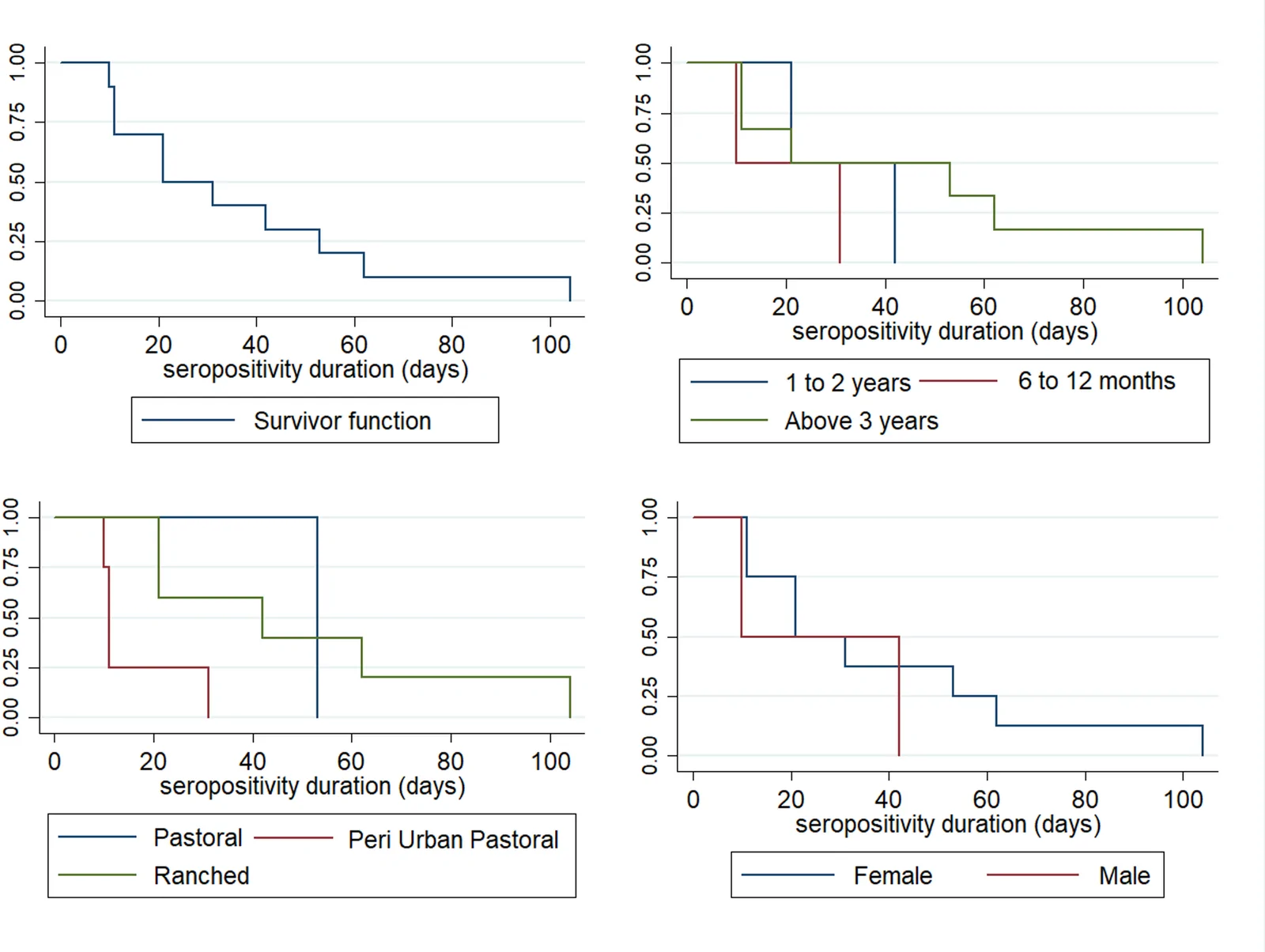
Kaplan-Meier plots showing the mean duration in days of seropositivity (**a**) and classified according to age (**b**), production system (**c**) and gender (**d**)

The median duration of seropositivity for the 30 uncensored episodes was 21 days (IQR: 11–53) (Table 4). Figure 2 illustrates these episodes grouped by age and censoring status. A multivariate Cox proportional hazards model was initially fitted to assess factors associated with seropositivity duration. However, the proportional hazards assumption was violated for the geographical site variable (global Schoenfeld residual test, *p* < 0.05). Consequently, a stratified Cox model was applied, with stratification by geographical site to account for non-proportional hazards. In this model, age and gender were not significant predictors of seropositivity duration. The extended durations observed in Garissa and Soysambu relative to Isiolo (2021) should be interpreted within the context of the stratified analysis, acknowledging the underlying heterogeneity across sites. The survival curves for these analyses are presented in Fig. 3; Table 4.

**Table 4:** Summary of median seropositivity durations

Variable	Category	Median Duration (Days)	Inter Quartile Range
Overall		21	11–53
Sex	Female	21	11–53
	Male	10	10–42
County	Garissa	53	53–53
	Soysambu (Nakuru)	42	21–62
	Isiolo (2018)	11	11–31
Production system	Pastoral (Free-range)	53	53–53
	Ranched	42	21–62
	Peri-urban	11	11–31

### RT-PCR and ppNT results for samples collected in Garissa County

**Fig. 4:**
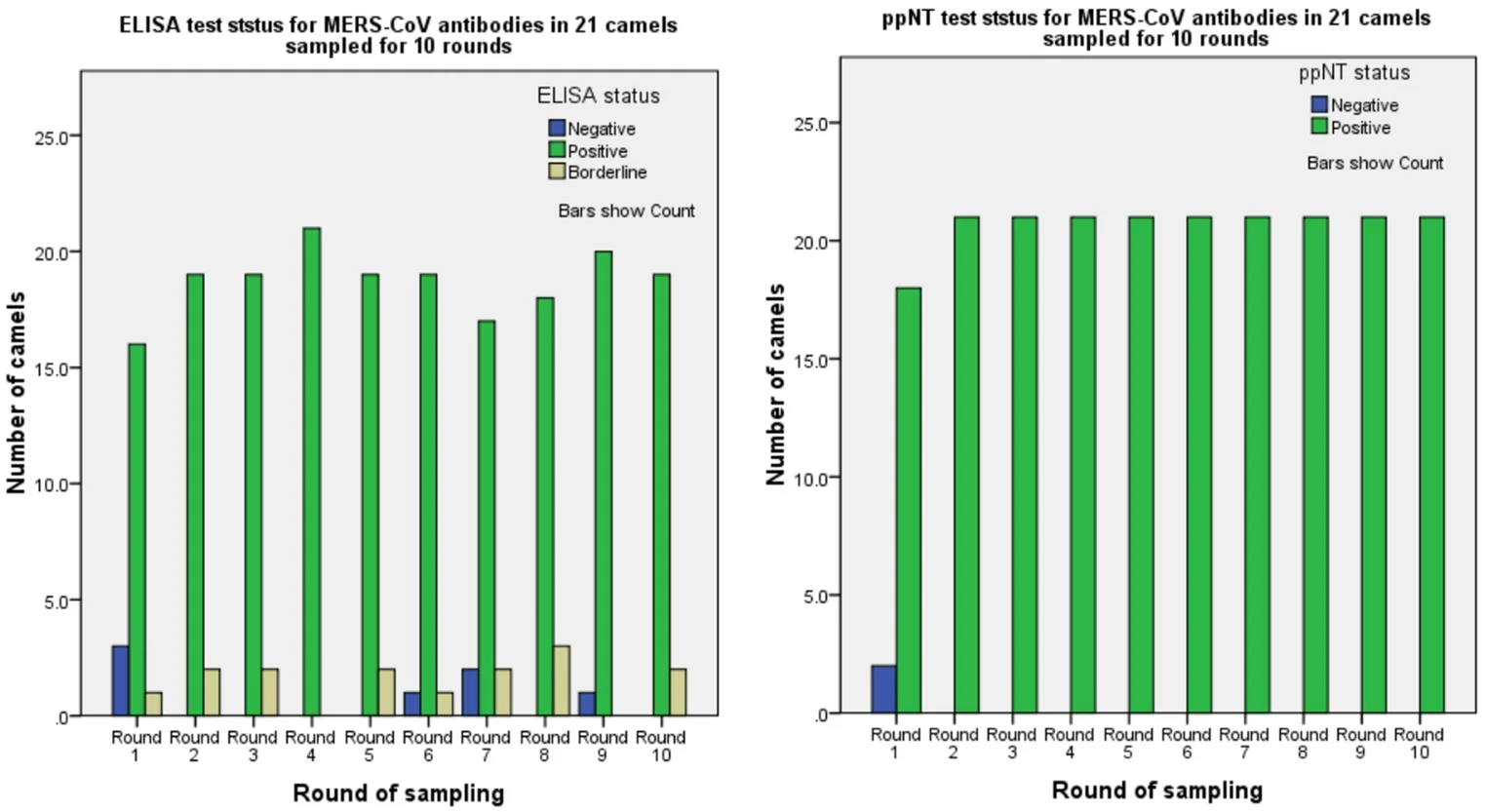
Comparative overall seropositivity for MERS-CoV of the Garissa cohort camels during the 2019 study period

Further analysis of the highly infected Garissa pastoral herd confirmed active virus shedding and validated the serological findings. Using the plaque reduction neutralisation test (ppNT) as a gold standard, the ELISA test demonstrated high sensitivity (97.4%) and specificity (100%), with a significant agreement between the two tests (Cohen’s k = 0.435, *p* < 0.0001). Figure 4 shows the seroconversion of the entire herd by the second sampling round.

Active MERS-CoV infection was confirmed via RT‑qPCR in nasal swabs from two adult female camels, yielding an RNA detection rate of 1% (2/209 samples) (Table 5). The cycle threshold (CT) values for the upE gene were 34.0 and 36.0. An additional six samples provided inconclusive RT‑qPCR results.

**Table 5:** RT-qPCR test outcomes by camel and round of sampling during the cohort study in Isiolo in 2019

Name of camel	Age	sex	CT value (upE gene)	RT-qPCR positivity		Round of sampling
				Confirmed	Inconclusive	
**Gaf**	Adult	Female	36.0	Yes	No	4
**Magor**	Adult	Female	34.0	Yes	No	6
Adhey Calf	Neonate	Female	38.0	No	Yes	10
Afgor	Adult	Female	37.0	No	Yes	4
Gaf 2	Adult	Female	42.0	No	Yes	8
Gafgredi	Adult	Female	38.0	No	Yes	4
Shidho	Adult	Female	37.0	No	Yes	5
Warai	Adult	Female	37.0	No	Yes	4

Results are reported at the animal level (per camel) in this case

**Fig. 5:**
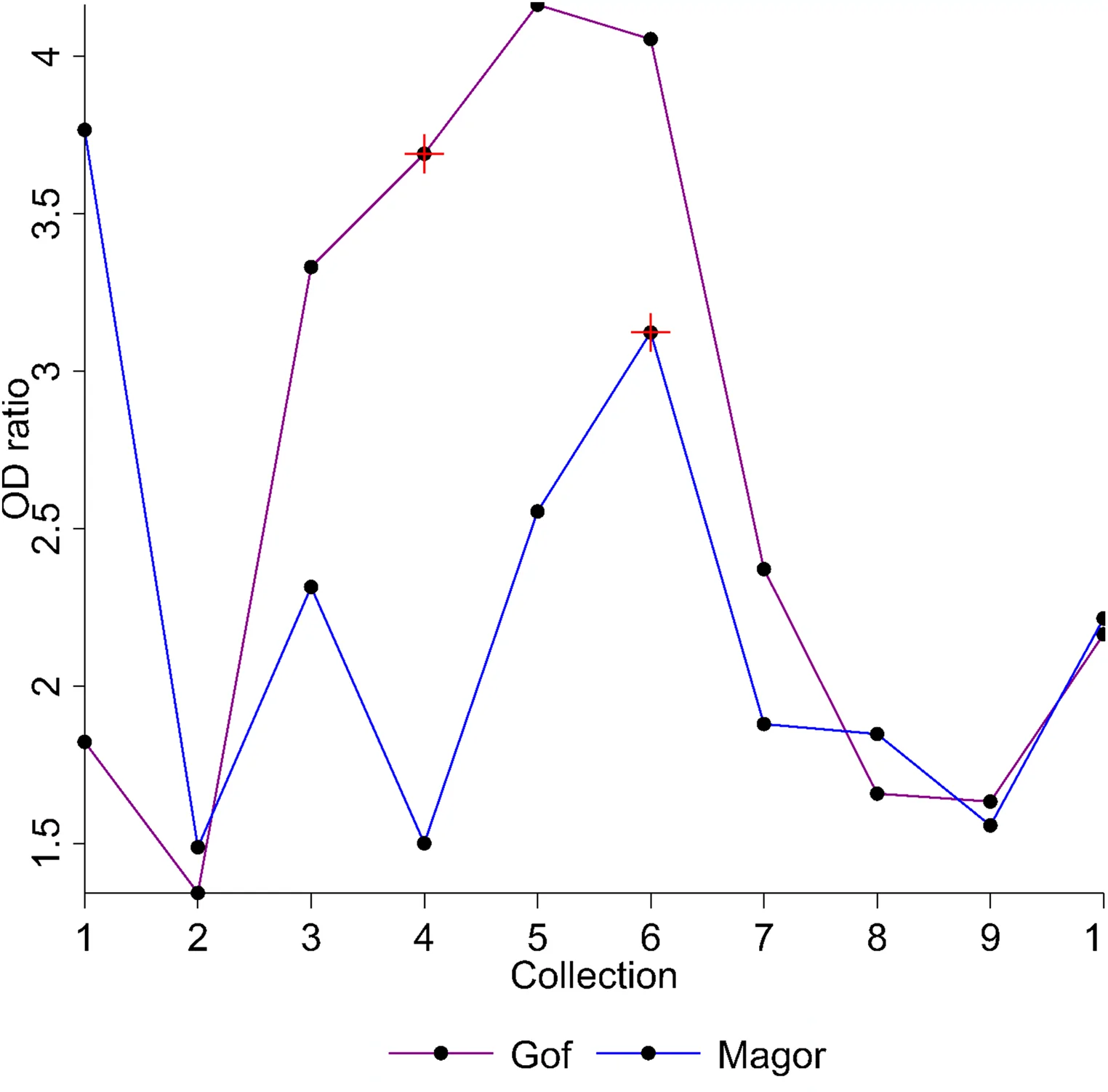
ELISA OD ratios plotted of two camels in which MERS-CoV RNA was detected plotted against round of sampling. PCR positive samples are shown with the ‘+’ sign

A comparison of PCR results with antibody dynamics showed that viral RNA detection coincided with seroconversion. Figure 5 plots the ELISA OD ratios for the two PCR-positive camels, Gaf and Magor. In both cases, more than a two-fold increase in antibody levels was observed. In Gaf, MERS-CoV RNA was detected about two weeks before the peak ELISA OD ratio, while in Magor, it was detected at the peak. Viral RNA was detected in two adult female camels coincident with rising ELISA OD ratios (Fig. 5). It should be noted that without sequencing, these findings cannot distinguish between reinfection, persistent infection, or prolonged shedding.

## Discussion

This study provides the first longitudinal characterization of MERS-CoV antibody kinetics and shedding dynamics in dromedary camels across different production systems in Kenya. Our major contribution is demonstrating that camel management practices are a primary determinant of MERS-CoV exposure and transmission dynamics, with significant implications for zoonotic risk assessment. The findings reveal that high seroprevalence in pastoral systems does not imply sterilizing immunity and that reinfection occurs.

The near-universal seroconversion (100%) observed in the pastoral production system in Garissa, compared to the minimal circulation (21.6% of individuals) in the ranched system in Soysambu, underscores the critical role of husbandry practices. Pastoralist dynamics, characterized by seasonal movements over vast ranges and constant mixing of herds at shared grazing areas and water points, create optimal conditions for viral transmission and maintenance (Gikonyo et al. 2018). In contrast, the closed, managed nature of the ranching system limits contact with outside herds, effectively reducing introduction and spread of the virus. This aligns with previous cross-sectional surveys in Kenya that reported seropositivity of 77.7% in pastoral systems versus 14.0% in ranches (Sitawa et al. 2020).

As expected, age was a significant predictor of seropositivity, with camels over three years having 21 times higher odds of being seropositive compared to newborns. However, the short duration of seropositivity in calves—often within a single sampling interval—likely reflects the decay of maternal antibodies before the development of a mature immune response (Meyer et al. 2016; Tang et al. 2020). More critically, the detection of viral RNA in adult camels concurrently with rising antibody titres suggests that prior exposure and seropositivity do not confer sterilizing immunity. This suggests that sterilizing immunity may be incomplete, allowing for recurrent viral detection, though the nature of these events requires virological confirmation. (Hemida et al. 2017; Farag et al. 2015).

A key finding with direct public health implications is the detection of active viral shedding via RT‑PCR in two adult female camels from the Garissa herd at a time when their antibody titres were rising sharply. This suggests that seropositivity does not necessarily prevent viral shedding. While this raises concerns about potential zoonotic transmission, our study did not include human serological or behavioral data, and thus we cannot directly quantify spillover risk. Phylogenetic evidence from Kenya suggests a history of undetected MERS-CoV spillover events (Stalin Raj et al. 2018), but the frequency and drivers of such events remain unclear and warrant targeted surveillance.

The significant geographical variation in seropositivity duration, with camels in Garissa and Soysambu having 97% and 98% longer durations than those in Isiolo, points to the influence of local environmental and ecological factors. While drought conditions during the study period may have increased herd congregation at limited water sources—potentially facilitating viral transmission—climatic variables were not formally analyzed in our models. This remains a postulated mechanism that should be investigated in future integrated studies (“Garissa_Drought_EWS_Bulletin_June_201920211215203640,” 2019a)-(“Garissa_Drought_EWS_Bulletin_September_201920211215203151,” 2019b). This spatiotemporal variability suggests that regional factors, such as climate stress and resource availability, can significantly modulate infection-reinfection dynamics beyond the core production system structure. We acknowledge perfect prediction in the pastoral system and the stark contrast with ranched systems remains epidemiologically informative. However, it is important to note that production system is confounded with geography and study phase, and caution should be exercised not to infer causal interpretation.

A key finding with direct public health implications is the detection of active viral shedding via RT‑PCR in two adult female camels from the Garissa herd at a time when their antibody titres were rising sharply. This temporal association suggests that seropositivity does not necessarily prevent viral shedding. However, without genomic sequencing or repeated PCR positivity, we cannot definitively distinguish between reinfection, prolonged viral shedding, or reactivation of latent infection. The rising antibody titres observed after PCR detection could reflect a primary infection in these animals rather than reinfection, a challenge commonly encountered when interpreting serological data in the absence of virological confirmation (Hemida et al. 2017; Farag et al. 2015). Thus, while our data indicate that prior exposure does not confer sterilizing immunity, the precise nature of the infection dynamics—whether reinfection, persistence, or reactivation—remains uncertain. This underscores the need for integrated virological and serological surveillance in longitudinal studies.

More critically, the detection of viral RNA in adult camels concurrently with rising antibody titres suggests that prior exposure and seropositivity do not preclude active viral replication. This indicates that sterilizing immunity against MERS-CoV may be limited and that recurrent viral activity occurs, a finding consistent with reports from Qatar and Saudi Arabia (Hemida et al. 2017; Farag et al. 2015). Nevertheless, we acknowledge that without sequencing data, we cannot rule out alternative explanations such as persistent low-level shedding or assay variability.

This work presented with certain limitations: The sampling schedule was inconsistent because herders moved their herds constantly and long period of follow-ups were practically impossible. The inconsistent pattern of the weather conditions made it difficult to relate periods to sampling months. In addition, some of the recruited camels were removed during the study period for various reasons. Furthermore, we excluded the left- and right-censored episodes, which may eliminate the majority of observed events, however we believed that the analytical choice was robust enough. Censored episodes were excluded only for median duration calculation to avoid bias from incomplete observation periods. The Kaplan–Meier and Cox models included censored data, as stated earlier. Though the duration estimates were based on uncensored episodes and should be interpreted with caution, survival analyses included all episodes. A similar approach had earlier been published in a longitudinal camel MERS-CoV study (Hemida et al. 2017). Additionally, the Cox proportional hazards model for seropositivity duration was limited by a small number of uncensored events, resulting in wide confidence intervals and potential model instability. Although we addressed the violation of proportional hazards through stratification, the findings should be interpreted with caution due to the limited sample size and inherent variability across study sites. Finally, the detection of viral RNA in only two animals, without accompanying genomic sequencing, limits our ability to distinguish between reinfection, persistent shedding, or reactivation. Although we observed a temporal coincidence between PCR positivity and rising antibody titres, this does not conclusively demonstrate reinfection and may reflect primary infection dynamics or assay variability.

This is our core One Health insight: high seroprevalence in pastoral herds should not be misinterpreted as protective herd immunity. Instead, it may reflect ongoing viral activity and a potential risk of spillover to humans in frequent contact with camels. However, the absence of reported human cases in Kenya underscores the need for coordinated human–animal surveillance to better understand transmission interfaces and risk factors.

## Conclusions and recommendations

Despite the limitations in this study, it establishes that management practices are a primary determinant of MERS-CoV exposure. The stark contrasts between production systems, combined with evidence of reinfection in seropositive adults. While our findings suggest that reinfection or recurrent shedding may occur in seropositive camels, the absence of genomic sequencing limits our ability to definitively characterize these events. Future studies incorporating viral sequencing and repeated molecular testing are needed to elucidate the mechanisms underlying recurrent MERS-CoV detection in seropositive animals. Tailored mitigation strategies, recognizing the distinct epidemiology in pastoral versus ranched systems, and considering the syndemic nature of these factors, are essential for effective MERS-CoV control and prevention of future spillover events.

## Supplementary Information

Below is the link to the electronic supplementary material.


Supplementary Material 1


## Data Availability

The datasets used and/or analysed during the current study are available on request.

## References

[CR9] Azhar EI, El-Kafrawy SA, Farraj SA, Hassan AM, Al-Saeed MS, Hashem AM et al. (2014) Evidence for camel-to-human transmission of MERS coronavirus. N Engl J Med 370(26):2499–2505. 10.1056/NEJMoa1401505 24896817

[CR333] EUROIMMUN, Medizinsche Labordiagnostika AG, (2015) New serological tests for an ongoing epidemic. MEDLAB Magazine 1:20–23. Available at: https://www.euroimmun.com/fileadmin/user_upload/News/Professional-articles/HI_2604_L_UK_A.pdf

[CR1] European Centre for Disease Prevention and Control (2025) https://www.ecdc.europa.eu/en/middle-east-respiratory-syndrome-coronavirus-mers-cov-situation-update. Accessed October 24 2025

[CR222] FAO (2019) Swab and tissue sample collection procedures enhancing MERS-CoV detection in camels: an illustrative guideline. Available at: https://openknowledge.fao.org/handle/20.500.14283/ca7428en. Accessed 15 November 2019

[CR2] Farag EABA, Chantal BEM, Reusken BL, Haagmans et al. (2015) High proportion of MERS-CoV shedding dromedaries at slaughterhouse with a potential epidemiological link to human cases, Qatar 2014. Infect Ecol Epidemiol 5(1). 10.3402/IEE.V5.28305 PMC4505336 26183160

[CR3] Garissa_Drought_EWS_Bulletin_June_201920211215203640 (2019a)

[CR4] Garissa_Drought_EWS_Bulletin_September_201920211215203151 (2019b)

[CR5] Gikonyo S, Kimani T, Matere J et al (2018) Mapping Potential Amplification and Transmission Hotspots. EcoHealth 15(2):372–387. 10.1007/s10393-018-1317-6 29549589 PMC7088189

[CR7] Hemida M, Gomaa A, Alnaeem, Daniel KW, Chu et al (2017) Longitudinal Study of Middle East respiratory syndrome coronavirus Infection in Dromedary Camel Herds in Saudi Arabia, 2014–2015. Emerg Microbes Infections 6(1):1–7. 10.1038/emi.2017.44 PMC5520318 28634355

[CR6] Hemida MG, Perera RA, Wang P et al (2013) Middle east respiratory syndrome (MERS) coronavirus seroprevalence in domestic livestock in Saudi Arabia, 2010 to 2013. Eurosurveillance 18(50). 10.2807/1560-7917.ES2013.18.50.20659 24342517

[CR8] Kelly-Cirino C, Mazzola LT, Chua A, Oxenford CJ, Kerkhove MDV (2019) An Updated Roadmap for MERS-CoV Research and Product Development: Focus on Diagnostics. BMJ Global Health 4(Suppl 2):e001105. 10.1136/bmjgh-2018-001105 30815285 PMC6361340

[CR10] Meyer B, Juhasz J, Barua R et al (2016) Time Course of MERS-CoV Infection and Immunity in Dromedary Camels. Emerg Infect Dis 22(12):2171–2173. 10.3201/eid2212.160382 27224315 PMC5189137

[CR11] Mohd HA, Jaffar A, Al-Tawfiq, Memish ZA (2016) Middle East respiratory syndrome coronavirus (MERS-CoV) Origin and Animal Reservoir. Virol J 13(1):87. 10.1186/s12985-016-0544-0 27255185 PMC4891877

[CR12] Munywoki PK, Dorothy C, Koech CN, Agoti et al (2014) The Source of Respiratory Syncytial Virus Infection In Infants: A Household Cohort Study. Rural Kenya J Infect Dis 209(11):1685–1692. 10.1093/infdis/jit828 24367040 PMC4017365

[CR13] Nyaguthii D, Machira GP, Otieno IK, Kombe et al (2021) Infection Patterns of Endemic Human Coronaviruses in Rural Households in Coastal Kenya. Wellcome Open Res 6:27. 10.12688/wellcomeopenres.16508.1 34957334 PMC8669777

[CR14] Park Y-S, Lee C, Kim KM et al (2015) The First Case of the 2015 Korean Middle East Respiratory Syndrome Outbreak. Epidemiol Health 37(November):e2015049. 10.4178/epih/e2015049 26725226 PMC4722220

[CR15] Perera RA, Wang P, Gomaa MR et al (2013) Seroepidemiology for MERS Coronavirus Using Microneutralisation and Pseudoparticle Virus Neutralisation Assays Reveal a High Prevalence of Antibody in Dromedary Camels in Egypt, June 2013. Eurosurveillance 18(36):1–7. 10.2807/1560-7917.ES2013.18.36.20574 24079378

[CR16] Risk Factors Predisposing Dromedary Camels and Pastoral Communities to Zoonotic.Pdf (2023)

[CR17] Sitawa R, Folorunso F, Obonyo M et al (2020) Risk factors for serological evidence of MERS-CoV in Camels, Kenya, 2016–2017. Prev Vet Med 185. 105197. 10.1016/j.prevetmed.2020.105197 PMC7605751 33186881

[CR18] Stalin Raj V, Nisreen MA, Okba J, Gutierrez-Alvarez et al (2018) Chimeric Camel/Human Heavy-Chain Antibodies Protect against MERS-CoV Infection. Sci Adv 4(8):eaas9667. 10.1126/sciadv.aas9667 30101189 PMC6082650

[CR444] StataCorp (2017) Stata Statistical Software: Release 15. College Station, TX: StataCorp LLC

[CR19] Tang L, Chen Y, Xiang Q, Xiang J, Tang Y, Li J (2020) The association between IL18, FOXP3 and IL13 genes polymorphisms and risk of allergic rhinitis: A meta-analysis. Inflamm Res 69(9):911–923. 10.1007/s00011-020-01368-4 32529476

[CR111] Younan M, Bornstein S, Gluecks IV (2016) MERS and the dromedary camel trade between Africa and the Middle East. Trop Anim Health Prod 48(6):1277–1282. 10.1007/s11250-016-1089-3 PMC7089074 27324244

[CR20] Zaki AM, Van Boheemen S, Bestebroer TM, Albert DME, Osterhaus, Ron AM, Fouchier (2012) Isolation of a Novel Coronavirus from a Man with Pneumonia in Saudi Arabia. N Engl J Med 367(19):1814–1820. 10.1056/NEJMoa1211721 23075143

