# Supplementary material for: Antibody kinetics and shedding dynamics of MERS-CoV in dromedary camels from different production systems in Kenya: a longitudinal cohort study
Source: Trop Anim Health Prod. 2026 Mar 12;58(2):169. doi: 10.1007/s11250-026-04981-3 (PMC12982281; doi:10.1007/s11250-026-04981-3)
Supplement: Supplementary file 1 — Supplementary Material 1 [file 11250_2026_4981_MOESM1_ESM.docx]

Additional comment.

The Table 5 is already mentioned in line 308 in the manuscript and the position is below the mention.
